# Eggplant latent viroid is located in the chloroplasts and nuclei of eggplant infected cells

**DOI:** 10.1186/s12985-024-02530-8

**Published:** 2024-10-15

**Authors:** Marcelo Eiras, Verónica Aragonés, Jorge Marqués, María Dolores Gómez, José-Antonio Daròs

**Affiliations:** 1 Lab. Fitovirologia e Fisiopatologia, Centro de Pesquisa de Sanidade Vegetal, Instituto Biológico, São Paulo, CEP 04014-002 SP Brazil; 2 Instituto de Biología Molecular y Celular de Plantas (Consejo Superior de Investigaciones Científicas-Universitat Politècnica de València), Valencia, 46022 Spain; 3 Azzur Group, Hatboro, USA

**Keywords:** Viroid, Non-coding RNA, Subcellular localization, *Avsunviroidae*, Elaviroid

## Abstract

Viroids that belong to genera *Avsunviroid* and *Pelamovirod* (family *Avsunviroidae*) replicate and accumulate in the chloroplasts of infected cells. In this report, we confirmed by RNA in situ hybridization using digoxigenin-UTP-labelled riboprobes that the positive strands of eggplant latent viroid (ELVd), the only member of genus *Elaviroid* within the family *Avsunviroidae*, also accumulate in the chloroplasts of infected cells. However, comparison of ELVd in situ hybridization signals with those from bona fide chloroplastic and nuclear non-coding RNAs, such as chloroplast 5S rRNA and U1 small nuclear RNA, supports the notion that this viroid is also present in the nuclei of infected cells. These results suggest that the subcellular localization of viroids within the family *Avsunviroidae* may be more complex than previously assumed with dynamic presence in several compartments during the infectious cycle.

**Supplementary Information:**

The online version contains supplementary material available at 10.1186/s12985-024-02530-8.

## Main text

Viroids are subviral infectious agents consisting of a single-stranded, circular, non-protein-coding RNA of approximately 250–450 nucleotides (nt). To complete the infectious cycle, viroids must recruit host enzymes and other factors to mediate replication and intracellular and intercellular traffic [1–3]. In addition, viroid RNA molecules need to shelter from host defensive responses to infection, notably RNA silencing [4–6]. Viroids must have adapted to host defense with some counter-defensive strategies that may include circular compact molecules with a high degree of self-complementarity [7], binding to cellular proteins [8, 9], interference with host transcription [10] or with host methylation [11]. Localization of viroid RNAs in sites in which RNA silencing is not particularly active, or is even absent, may also contribute to shelter viroids from RNA silencing.

Viroids that belong to the family *Avsunviroidae* [12, 13] replicate through a symmetric rolling-circle mechanism mediated by host enzymes and the hammerhead ribozymes embedded in the RNA molecules of both polarities [14–18]. A series of experimental evidences support that viroids belonging to this family replicate and accumulate in the chloroplasts of infected plants. They include direct detection of the RNA of avocado sunblotch viroid (ASBVd, the type member of the genus *Avsunviroid* and the family *Avsunviroidae*) and peach latent mosaic viroid (PLMV, type member of the genus *Pelamoviroid*) inside the chloroplasts of infected cells by in situ hybridization [16, 19, 20]. This notion is also supported by the chloroplast localization of the enzymes likely involved in ASBVd RNA transcription, a chloroplastic nuclear-encoded DNA-dependent RNA polymerase (NEP) [17], and the chloroplastic isoform of tRNA ligase involved in eggplant latent viroid (ELVd, genus *Elaviroid*, *Elaviroid latensmelongenae*) circularization [21]. In contrast, members of the family *Pospiviroidae* [22] that replicate through an asymmetric rolling-circle mechanism have been localized in the nuclei of infected cells [23, 24].

ELVd is the type and only member of the genus *Elaviroid* within the family *Avsunviroidae* [25, 26]. This viroid was assumed to replicate in the chloroplasts of infected cells by analogy to the other members of the family, although belonging to different genera. Supporting this assumption, analysis of RNA trafficking using fluorescent reporter genes indicated the ability of ELVd RNA to translocate into the chloroplasts [27, 28], although further analysis suggested a more complex pathway that may involve a nuclear step [29, 30]. To confirm ELVd subcellular localization in eggplant infected cells, in this work we used in situ hybridization with digoxigenin (DIG)-UTP-labelled riboprobes to determine the accumulation site of the ELVd strands of plus polarity.

**Fig. 1:**
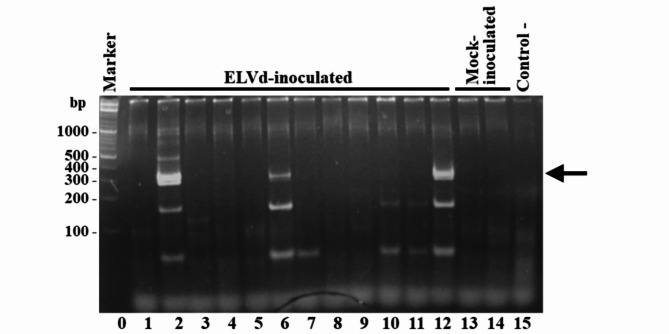
RT-PCR analysis of ELVd infection in eggplants. Plants were mechanically inoculated with ELVd RNA. The presence of ELVd RNA in upper non-inoculated tissues was analyzed one month after inoculation by RT-PCR followed by PAGE analysis of the products. Lane 0, DNA ladder marker with size of some of the molecules (in bp) on the left; lanes 1 to 12, ELVd-inoculated eggplants; lanes 13 and 14, mock-inoculated eggplants; lane 15, RT-PCR negative control with no template added. Arrow on the right points the 333-bp ELVd cDNA amplified in samples corresponding to infected eggplants

To investigate whether ELVd, as other viroids that are members of the family *Avsunviroidae*, accumulates in the chloroplasts of infected cells, we planned to subject tissues of ELVd-infected eggplants to in situ hybridization with a viroid complementary DIG-labelled RNA probe. First, we mechanically inoculated 12 eggplants (*Solanum melongena* L.; cultivar Black Beauty) using circular ELVd produced in *E. coli* [31]. Inoculation was performed gently distributing the RNA preparation in 5% carborundum, 50 mM K_2_HPO_4_ on the adaxial side of a leaves using a 3-mm-diameter glass rod. Plants were grown in a greenhouse at 25ºC with a 16/8 h day/night cycle. Upper non-inoculated leaf tissues were collected 1 month post-inoculation and RNA purified using silica columns (Zymo Research). ELVd infection was diagnosed by reverse transcription (RT) using oligonucleotide primer D186 (5’-GTGGCACACACCACCCTATGG-3’; complementary to positions 329 to 333 and 1 to 16 of ELVd-AJ536613; note that ELVd is a circular RNA and nt 333 is followed with nt 1), followed by polymerase chain reaction (PCR) using oligonucleotide primers D186 and D187 (5’-CCCTGATGAGACCGAAAGGTC-3’; homologous to ELVd-AJ536613 positions 17 to 36). PCR products were separated by polyacrylamide gel electrophoresis (PAGE) in a 5% polyacrylamide [39:1 acrylamide: *N*,* N’*-methylene (bisacrylamide)] gels in buffer TAE (40 mM Tris, 20 mM sodium acetate, 1 mM EDTA, pH 7.2). Gels were stained for 15 min in 1 µg/ml ethidium bromide. ELVd RT-PCR diagnosis indicated that only 3 out of the 12 plants were infected (Fig. 1). No symptoms were detected in the infected plants as previously reported [26, 32, 33]. The relatively low infection rate (25%) supported eggplant recalcitrant reaction to ELVd mechanical inoculation, possibly related to the robust morphology of eggplant leaves. The infected plants one month after inoculation, as well as equivalent mock-inoculated controls, were selected for ELVd subcellular localization analysis by in situ hybridization.

Leaf tissue sections from the mock-inoculated and the ELVd-infected eggplants were examined under the differential interference contrast (DIC) microscope after hybridization with RNA probes. DIG-labelled RNA probes were produced by in vitro transcription using T3 RNA polymerase in the presence of 5 mM DIG-11-UTP (Roche). RNA probe sequences were complementary to ELVd (AJ536613), eggplant chloroplast 5S rRNA and U1 small nuclear RNA (Supplementary Fig. S1). Leaf pieces from mock-inoculated and ELVd-infected eggplants were fixed in solution FAE (50% ethanol, 10% formaldehyde, 5% acetic acid) and embedded in paraffin using an automatic tissue processor TP1020 (Leica Biosystems). Microtome sections (8 μm) were deparaffinized with Histoclear, subjected to acid hydrolysis (0.2 M HCl for 20 min) at room temperature and proteinase K digestion (1 µg/ml in 10 mM Tris-HCl, 50 mM EDTA pH 8.0 for 15 min at 37ºC). Tissue sections were next fixed in 4% formaldehyde in phosphate buffered saline (PBS) solution for 10 min. Tissue preparations were subjected to prehybridization and hybridization for 1 and 12 h, respectively, at 54ºC inside a humidified plastic box. Hybridization buffer consisted of 50% formamide in SSC (150 mM NaCl, 15 mM sodium citrate, pH 7.0). DIG-labelled probes were detected using an anti-DIG antibody (Roche) conjugated to alkaline phosphatase and chromogenic substrates (4-nitro blue tetrazolium chloride –NBT– and 5-bromo-4-chloro-3-indolyl phosphate p-toluidine salt –BCIP–). After a 12 h incubation, tissues were observed under a DIC microscope (Nikon Eclipse E600). In order to discern the specific subcellular localization of ELVd, we compared hybridization signals of the ELVd, chloroplast 5S rRNA and U1 snRNA riboprobes by merging these images with those obtained from the same leaf tissues after staining with the nuclei specific dye 4’,6-diamidino-2-phenylindole dihydrochloride (DAPI), and examining under UV light at DIC microscope.

**Fig. 2:**
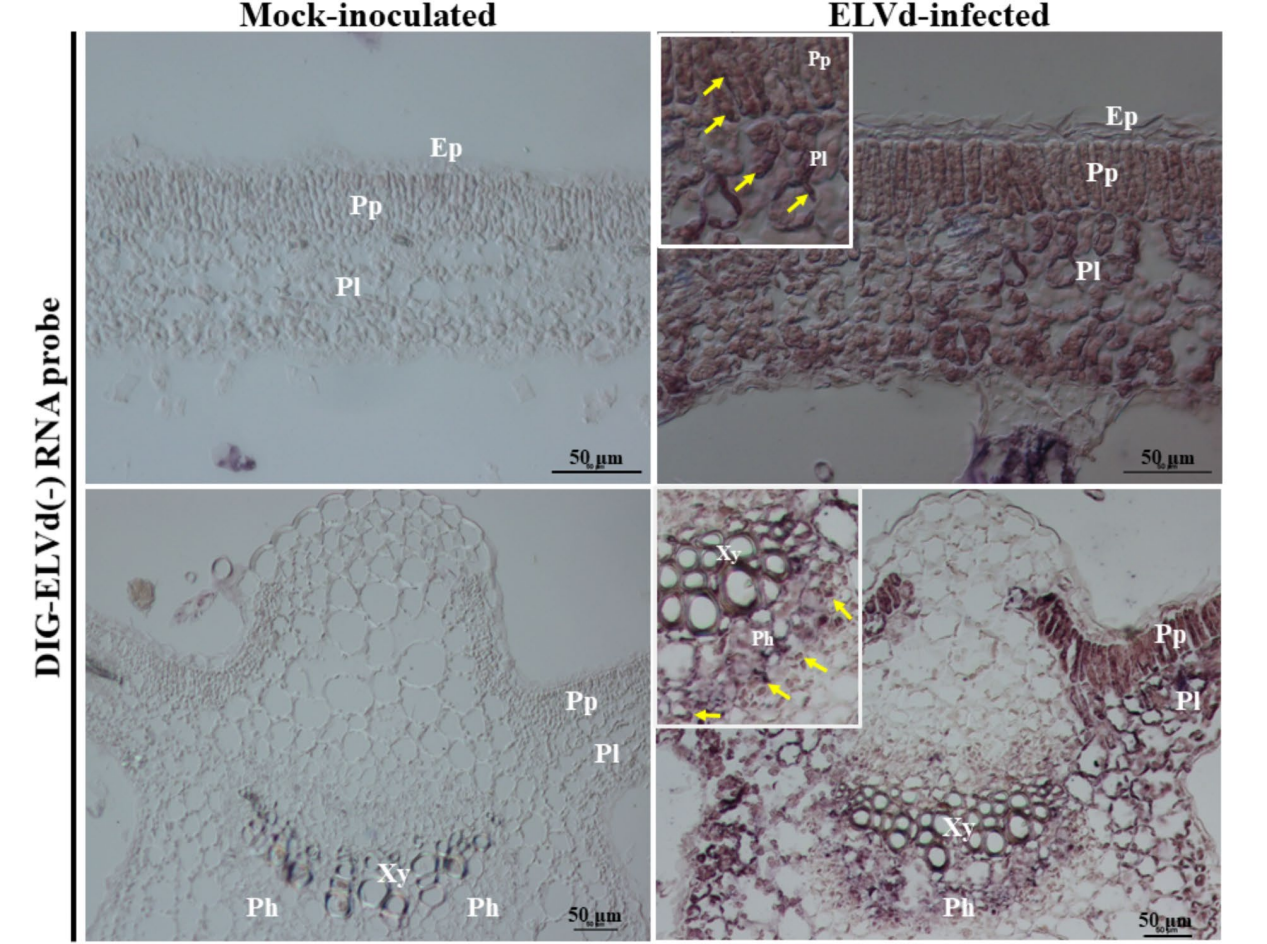
In situ hybridization of mock-inoculated (left images) and ELVd-infected (right images) eggplant tissues with an ELVd DIG-labelled probe of minus polarity. Representative DIC microscope images are shown. Arrows, in detail insets, indicate hybridization signals in chloroplasts of the palisade and lacunar parenchyma (upper right image), and phloem cells (lower right image). Ep, epidermis; Pp and Pl, palisade and lacunar parenchyma, respectively; Ph, phloem; Xy, xylem. Bars represent 50 μm, as indicated

After colorimetric staining, while no substantial hybridization signals were observed in sections from mock-inoculated plants, strong signals were observed in all sections from infected eggplants. Representative sections are shown in Fig. 2 (compare left with right pictures corresponding to mock-inoculated and ELVd-infected plants, respectively). In tissues infected with ELVd, hybridization signals localized in chloroplasts of both palisade and lacunar parenchyma cells, as well as those from phloem cells (Fig. 2, pictures on the right). Infected tissues showed rather homogeneous hybridization signals, i.e., they were distributed throughout the whole leaf blade. This indicates an even colonization of the infected plants, which is in agreement with a previous observation indicating that ELVd was uniformly distributed in leaves, stems and fruits (skin and pulp) [33].

**Fig. 3:**
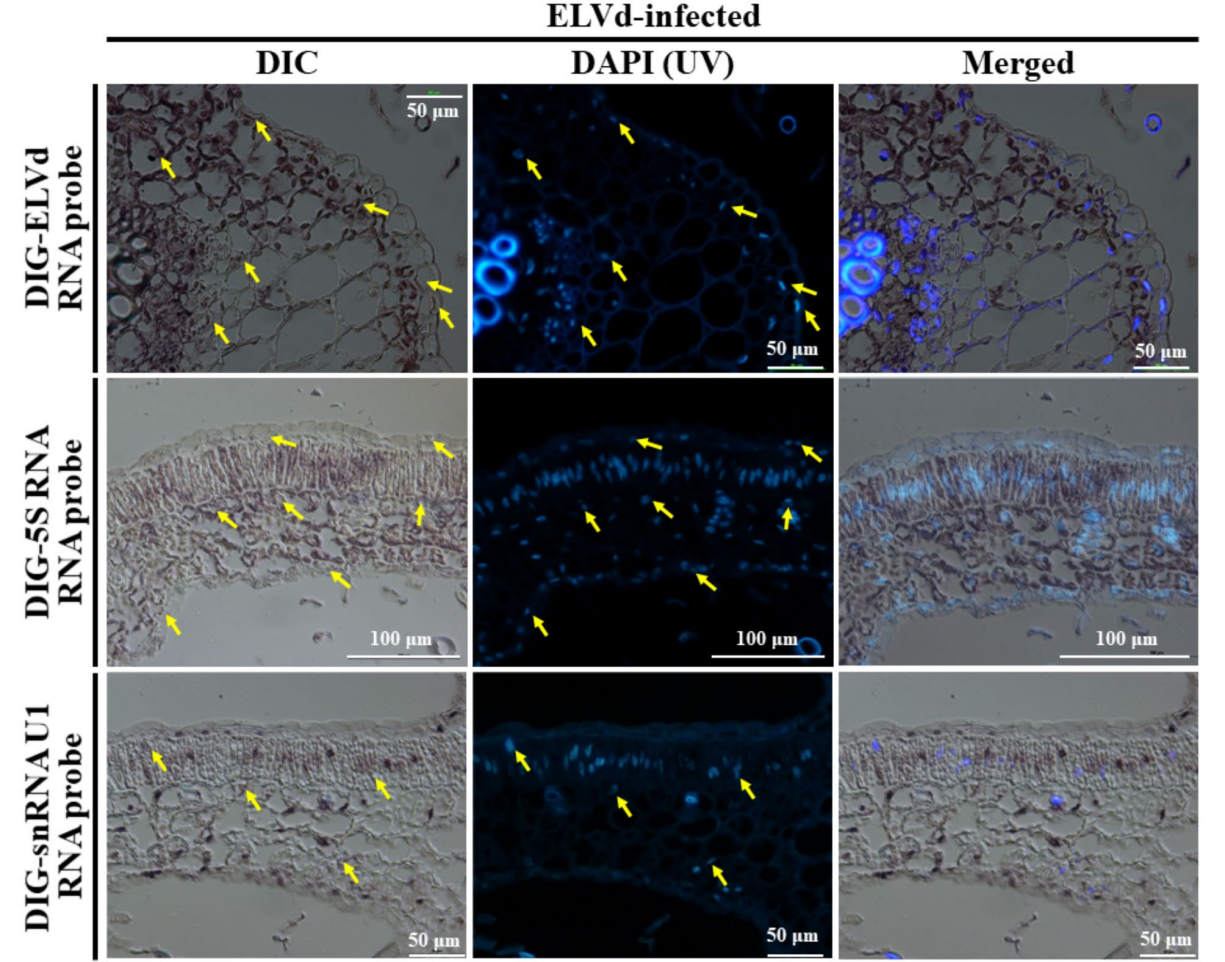
In situ hybridization of ELVd-infected eggplant tissues with DIG-labelled RNA probes complementary to ELVd (upper row), and eggplant chloroplast 5S rRNA (central row) and snRNA U1 (lower row). Representative DIC microscope images are shown on the left column. After reveling in situ hybridization, sections were subjected to DAPI staining (images on the central column). Merged images are shown on the right column. Arrows point to some representative nuclei that are stained with DAPI and also show hybridization signal from the ELVd and snRNA U1 probes, but not from that corresponding to chloroplast 5S rRNA. Merged images were obtained with Fiji IJ software. Bars represent 50–100 μm, as indicated

Careful inspection of the sections also suggested the ELVd RNA presence in the nuclei of infected cells (Fig. 2). To further investigate this notion, we performed a comparative localization analysis of ELVd RNA and two eggplant endogenous small non-coding RNAs, namely chloroplastic 5S rRNA and snRNA U1, that are well-known to accumulate in chloroplasts and nuclei, respectively. Sections from infected eggplants were hybridized with DIG-labelled RNA probes complementary to ELVd, 5S rRNA and snRNA U1. This analysis also included section staining with DAPI to better identify nuclei. In contrast to hybridization signals obtained with the probe complementary to 5S rRNA, signals from the snRNA U1 and ELVd complementary probes and those from DAPI staining highlighted similar round organelles likely corresponding to nuclei of infected cells (Fig. 3). Taken together these results indicate that plus-strand ELVd RNAs accumulate in the chloroplasts, but also in the nuclei of infected cells.

## Discussion

Viroids that belong to the family *Avsunviroidae* are considered to replicate and accumulate in the chloroplast of infected cells, opposite to those that belong to the family *Pospiviroidae* that are proposed to replicate and accumulate in nuclei [3]. Because these general statements arise from analyses with a limited number of viroid species and viroid subcellular trafficking in infected cells could be a rather complex dynamic process, in this work we aimed to analyze the subcellular localization in infected eggplant tissues of the plus RNA strands of ELVd, the only member of the genus *Elaviroid* within the family *Avsunviroidae*.

Our experimental results clearly showed that ELVd accumulates in the chloroplasts (Fig. 2), but they also indicated that a minor fraction accumulates in the nuclei of infected cells. This second observation is based on differential accumulation of bona fide chloroplast and nuclear non-coding RNAs endogenous to eggplant cells (Fig. 3). Previous in situ hybridization analyses showed that some members of the family *Pospiviroidae*, such as potato spindle tuber viroid (PSTVd), coconut cadang-cadang viroid (CCCVd) and citrus exocortis viroid (CEVd) accumulate in the nuclei of infected cells [34, 35], although at least in the case of PSTVd, RNAs of plus and minus polarities exhibit differential subnuclear localization [23]. In contrast, some members of the family *Avsunviroidae*, namely ASBVd and PLMVd, were shown to accumulate in the chloroplast of infected cells [16, 19, 20]. Evidence that ELVd was also associated with chloroplasts was anticipated by Gómez and Pallás (2010a; 2010b), based on confocal laser scanning microscopy observations of *N. benthamiana* tissues infiltrated with *Agrobacterium tumefaciens* clones transformed with constructs in which a green fluorescent protein (GFP) cDNA was fused to ELVd sequences. These studies raised the possibility of a subcellular signaling pathway, still unknown in plants, regulating selective import of RNA molecules into chloroplasts. ELVd, like other members of the family *Avsunviroidae*, may have taken advantage of this pathway to gain access to the chloroplast environment for replication and accumulation [27, 28]. Chloroplast may constitute a subcellular compartment in which the partially double-stranded strands of viroid progeny, and particularly the perfect double-stranded RNA viroid replication intermediates, are sheltered from the host defensive RNA silencing pathways. Plastids are considered organelles in which the double-stranded RNA processing machinery is absent [36]. This is the reason why high-level accumulation and efficacy of long insecticidal double-stranded RNAs only occurs when expressed in plastids [37, 38].

The possibility that ELVd also locates in the nuclei of infected eggplant cells was also suggested by Gómez and Pallás (2012a and 2012b), based on expression in *N. benthamiana* of potato virus X (PVX) constructs with an intronized GFP that only produced fluorescence when PSTVd or ELVd sequences were also inserted. Results suggested that sequences of both viroids contributed to intron removal, likely after being subjected to the nucleus mRNA splicing machinery [29, 30]. Interestingly, previous cell fractionation analyses with ASBVd-infected avocado tissues based on differential centrifugation showed that small amounts of ASBVd RNAs were also recovered from the nuclear fraction [39, 40]. Similarly, in situ hybridization experiments with PLMVd-infected tissues under transmission electron microscope localized colloidal gold particles predominantly in the chloroplasts, but hybridization signals were also observed in the nuclei [16].

Our experimental results here, in conjunction with previous observations, support that subcellular accumulation of ELVd may definitively be more complex than initially presumed and encompass several subcellular locations, including chloroplasts and nuclei. This may also occur with the other members of the family *Avsunviroidae*.

## Electronic supplementary material

Below is the link to the electronic supplementary material.

Supplementary Material 1

## Data Availability

Data is provided within the manuscript or supplementary information files.
