# Supplementary material for: Eggplant latent viroid is located in the chloroplasts and nuclei of eggplant infected cells
Source: Virol J. 2024 Oct 15;21:254. doi: 10.1186/s12985-024-02530-8 (PMC11476940; doi:10.1186/s12985-024-02530-8)
Supplement: Supplementary file 1 — Supplementary Material 1 [file 12985_2024_2530_MOESM1_ESM.pdf]

SUPPLEMENTARY MATERIAL

Eggplant latent viroid is located in the chloroplasts and nuclei of eggplant infected cells

Marcelo Eiras[1,2*], Verónica Aragonés[2], Jorge Marqués[2,3], María Dolores Gómez[2], José-Antonio Daròs[2*]

*Correspondence:
Marcelo Eiras
marcelo.eiras@sp.gov.br
José-Antonio Daròs
jadaros@ibmcp.upv.es

[1]Lab. Fitovirologia e Fisiopatologia, Centro de Pesquisa de Sanidade Vegetal, Instituto Biológico, São Paulo, SP, CEP 04014-002, Brazil
[2]Instituto de Biología Molecular y Celular de Plantas (Consejo Superior de Investigaciones Científicas-Universitat Politècnica de València), 46022 Valencia Spain
[3]Current address: Azzur Group, USA

Fig. S1. cDNA sequences of ELVd and eggplant chloroplast 5S rRNA and snRNA U1 cloned in reverse orientation to produce DIG-labelled RNA probes for *in situ* hybridization.

>ELVd (GeneBank accession no. AJ536613)
GGGTGGTGTGTGCCACCCCTGATGAGACCGAAAGGTCGAAATGGGGTTTCGCCATGGGTCGGGACTTTAAATT
CGGAGGATTCGTCCTTTAAACGTTCCTCCAAGAGTCCCTTCCCCAAACCCTTACTTTGTAAGTGTGGTTCGGC
GAATGTACCGTTTCGTCCTTTCGGACTCATCAGGGAAAGTACACACTTTCCGACGGTGGGTTCGTCGACACCT
CTCCCCCTCCCAGGTACTATCCCCTTTCAAGGATGTGTTCCCTAGGAGGGTGGGTGTACCTCTTTTGGATTGC
TCCGGCCTTCCAGGAGAGATAGAGGACGACCTCTCCCCATA

>Chloroplast 5S rRNA eggplant
GATCATCCTGGCGTCGAGCTATTTTTCCGCAGGACCTCCCCTACAGTATCGTCACCGCAGTAGAGT
TTAACCACCAAGTTCGGGATGGATTGGTGTGGTTCCTCTACGCCTAGGACACCAGAATA

>snRNA U1 eggplant
TATACTTAGCTGGACGGGGTCAATGGATGATCAAGAAGGTTCATGGCCTAGGTTGGTGACTTCCATCGCACTT
TGGAGGGGTGTATGCCTAAGGTCAACCCAAGTGGTTGAGCCTATGTC
